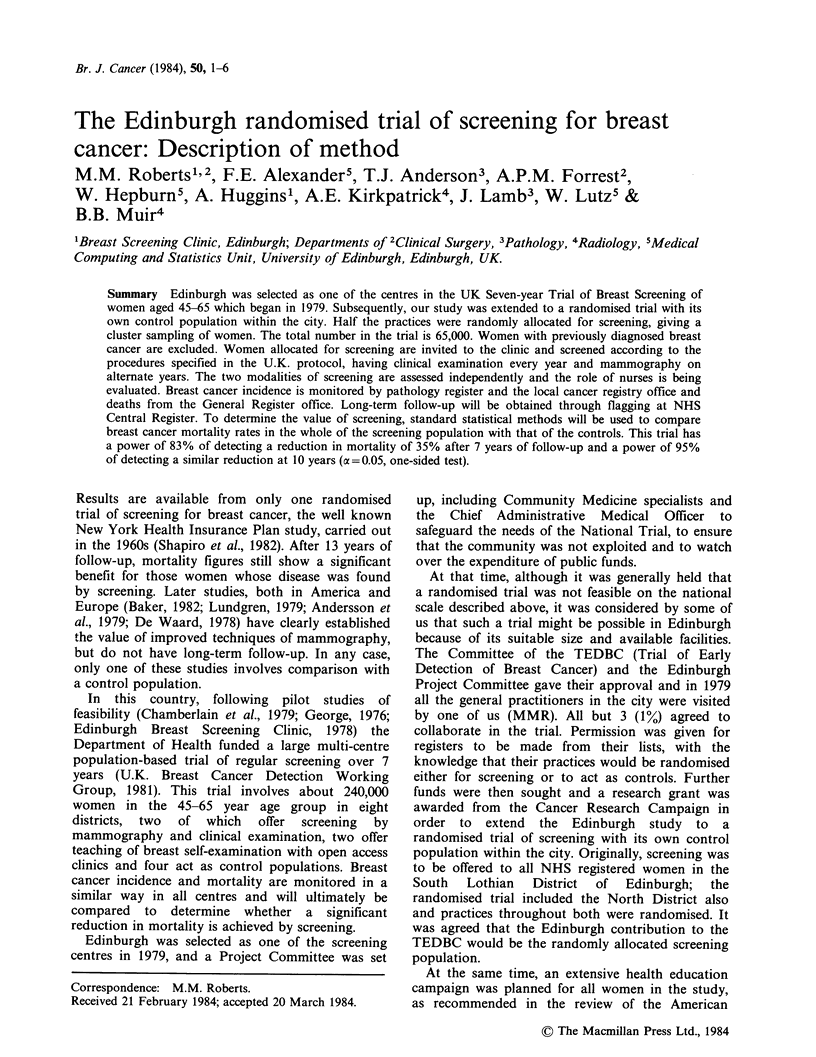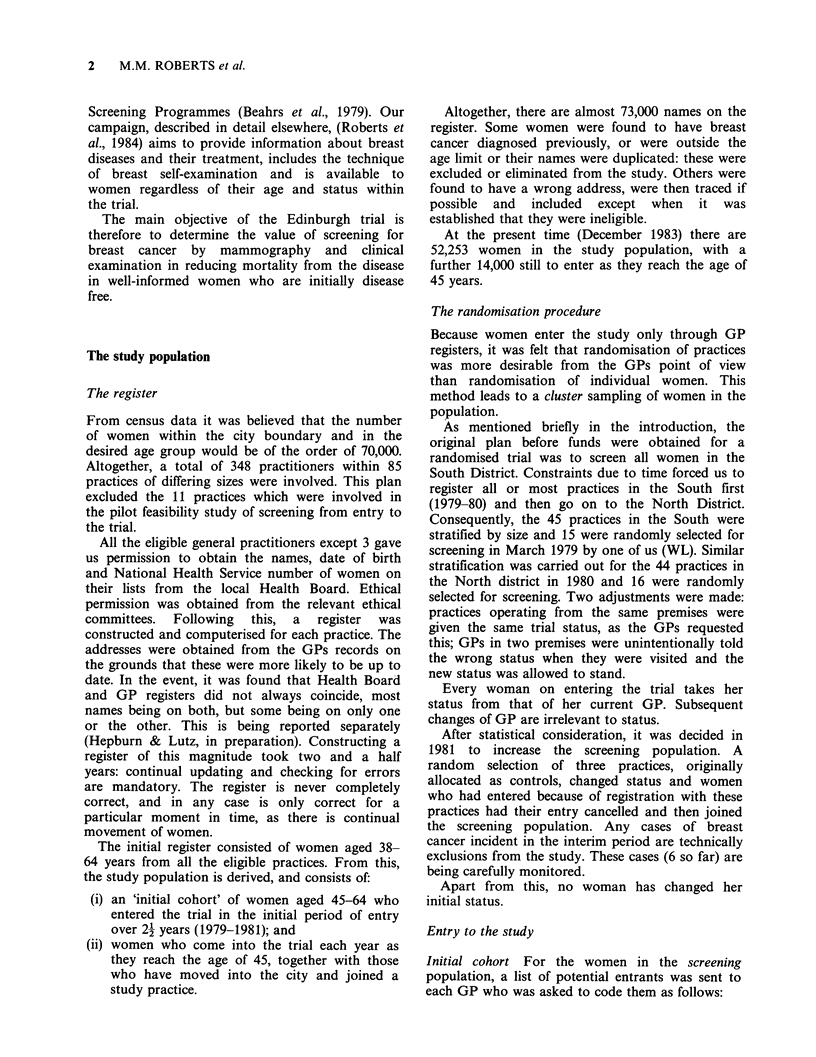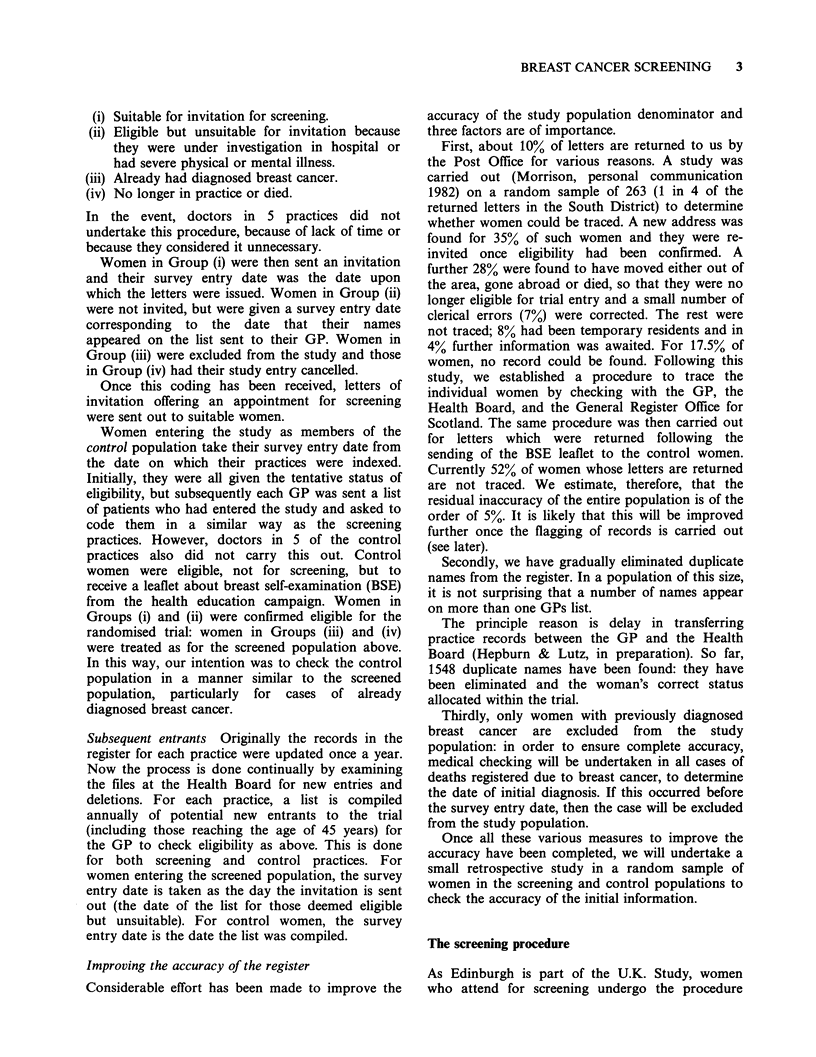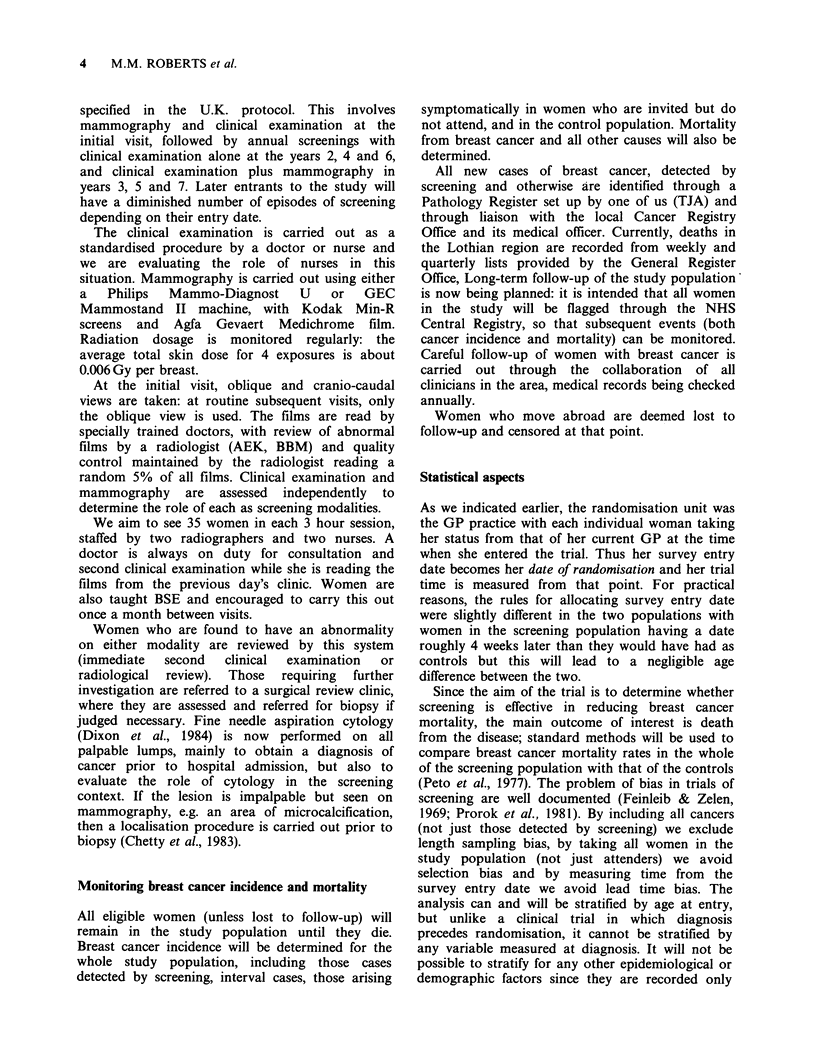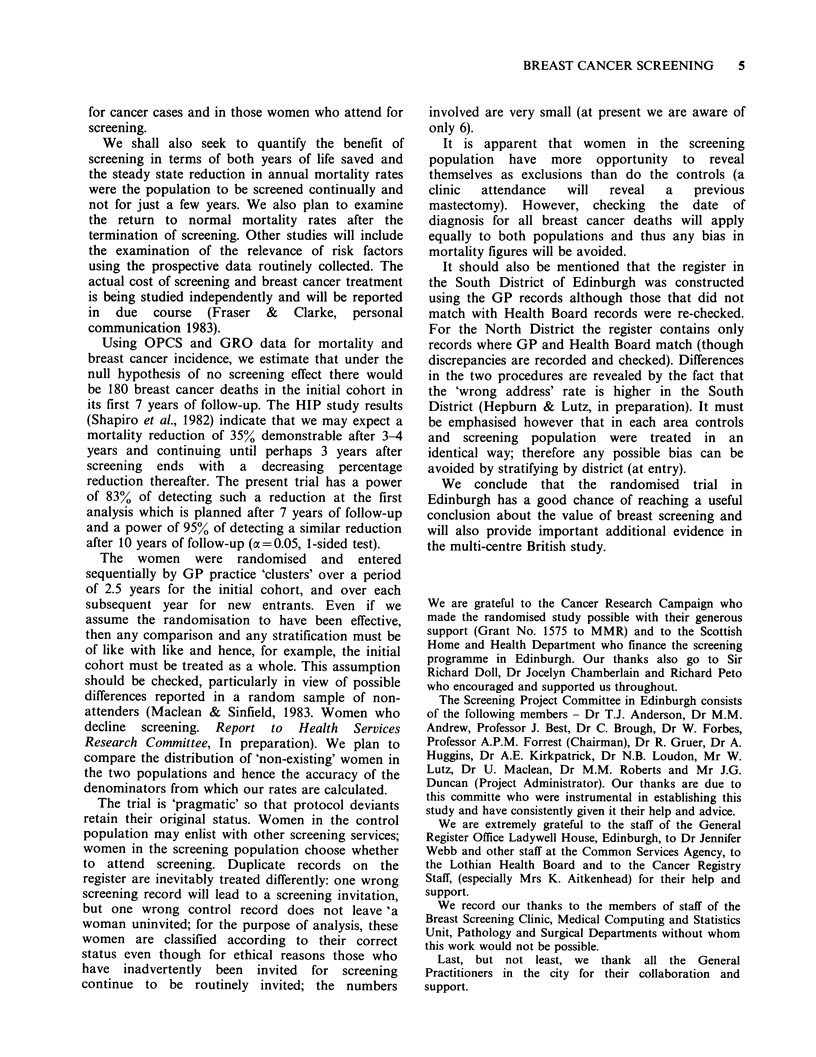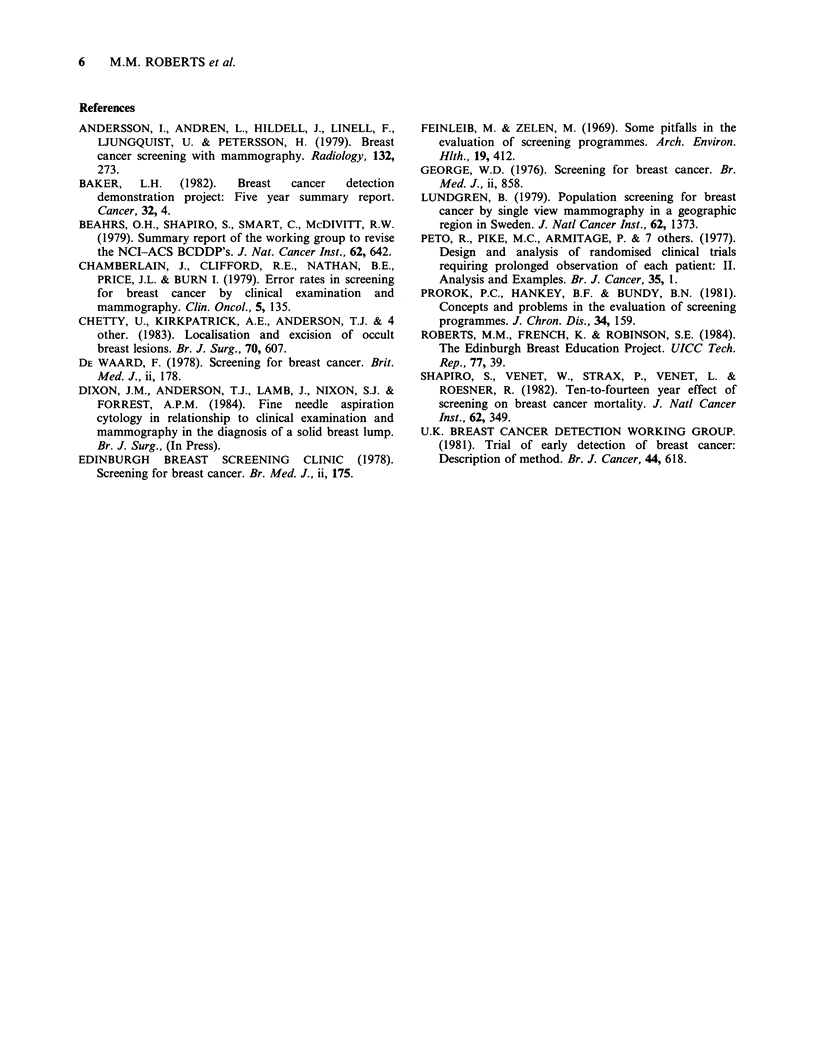# The Edinburgh randomised trial of screening for breast cancer: description of method.

**DOI:** 10.1038/bjc.1984.132

**Published:** 1984-07

**Authors:** M. M. Roberts, F. E. Alexander, T. J. Anderson, A. P. Forrest, W. Hepburn, A. Huggins, A. E. Kirkpatrick, J. Lamb, W. Lutz, B. B. Muir

## Abstract

Edinburgh was selected as one of the centres in the UK Seven-year Trial of Breast Screening of women aged 45-65 which began in 1979. Subsequently, our study was extended to a randomised trial with its own control population within the city. Half the practices were randomly allocated for screening, giving a cluster sampling of women. The total number in the trial is 65,000. Women with previously diagnosed breast cancer are excluded. Women allocated for screening are invited to the clinic and screened according to the procedures specified in the U.K. protocol, having clinical examination every year and mammography on alternate years. The two modalities of screening are assessed independently and the role of nurses is being evaluated. Breast cancer incidence is monitored by pathology register and the local cancer registry office and deaths from the General Register office. Long-term follow-up will be obtained through flagging at NHS Central Register. To determine the value of screening, standard statistical methods will be used to compare breast cancer mortality rates in the whole of the screening population with that of the controls. This trial has a power of 83% of detecting a reduction in mortality of 35% after 7 years of follow-up and a power of 95% of detecting a similar reduction at 10 years (alpha = 0.05, one-sided test).


*Br. J. Cancer* (1984), **50**, 1–6

### The Edinburgh randomised trial of screening for breast cancer: Description of method

M.M. Roberts[1,2], F.E. Alexander[5], T.J. Anderson[3], A.P.M. Forrest[2], W. Hepburn[5], A. Huggins[1], A.E. Kirkpatrick[4], J. Lamb[3], W. Lutz[5] & B.B. Muir[4]

[1]*Breast Screening Clinic, Edinburgh; Departments of* [2]*Clinical Surgery,* [3]*Pathology,* [4]*Radiology,* [5]*Medical Computing and Statistics Unit, University of Edinburgh, Edinburgh, UK.*

**Summary** Edinburgh was selected as one of the centres in the UK Seven-year Trial of Breast Screening of women aged 45–65 which began in 1979. Subsequently, our study was extended to a randomised trial with its own control population within the city. Half the practices were randomly allocated for screening, giving a cluster sampling of women. The total number in the trial is 65,000. Women with previously diagnosed breast cancer are excluded. Women allocated for screening are invited to the clinic and screened according to the procedures specified in the U.K. protocol, having clinical examination every year and mammography on alternate years. The two modalities of screening are assessed independently and the role of nurses is being evaluated. Breast cancer incidence is monitored by pathology register and the local cancer registry office and deaths from the General Register office. Long-term follow-up will be obtained through flagging at NHS Central Register. To determine the value of screening, standard statistical methods will be used to compare breast cancer mortality rates in the whole of the screening population with that of the controls. This trial has a power of 83% of detecting a reduction in mortality of 35% after 7 years of follow-up and a power of 95% of detecting a similar reduction at 10 years ($\alpha = 0.05$, one-sided test).

Results are available from only one randomised trial of screening for breast cancer, the well known New York Health Insurance Plan study, carried out in the 1960s (Shapiro *et al.*, 1982). After 13 years of follow-up, mortality figures still show a significant benefit for those women whose disease was found by screening. Later studies, both in America and Europe (Baker, 1982; Lundgren, 1979; Andersson *et al.*, 1979; De Waard, 1978) have clearly established the value of improved techniques of mammography, but do not have long-term follow-up. In any case, only one of these studies involves comparison with a control population.

In this country, following pilot studies of feasibility (Chamberlain *et al.*, 1979; George, 1976; Edinburgh Breast Screening Clinic, 1978) the Department of Health funded a large multi-centre population-based trial of regular screening over 7 years (U.K. Breast Cancer Detection Working Group, 1981). This trial involves about 240,000 women in the 45–65 year age group in eight districts, two of which offer screening by mammography and clinical examination, two offer teaching of breast self-examination with open access clinics and four act as control populations. Breast cancer incidence and mortality are monitored in a similar way in all centres and will ultimately be compared to determine whether a significant reduction in mortality is achieved by screening.

Edinburgh was selected as one of the screening centres in 1979, and a Project Committee was set up, including Community Medicine specialists and the Chief Administrative Medical Officer to safeguard the needs of the National Trial, to ensure that the community was not exploited and to watch over the expenditure of public funds.

At that time, although it was generally held that a randomised trial was not feasible on the national scale described above, it was considered by some of us that such a trial might be possible in Edinburgh because of its suitable size and available facilities. The Committee of the TEDBC (Trial of Early Detection of Breast Cancer) and the Edinburgh Project Committee gave their approval and in 1979 all the general practitioners in the city were visited by one of us (MMR). All but 3 (1%) agreed to collaborate in the trial. Permission was given for registers to be made from their lists, with the knowledge that their practices would be randomised either for screening or to act as controls. Further funds were then sought and a research grant was awarded from the Cancer Research Campaign in order to extend the Edinburgh study to a randomised trial of screening with its own control population within the city. Originally, screening was to be offered to all NHS registered women in the South Lothian District of Edinburgh; the randomised trial included the North District also and practices throughout both were randomised. It was agreed that the Edinburgh contribution to the TEDBC would be the randomly allocated screening population.

At the same time, an extensive health education campaign was planned for all women in the study, as recommended in the review of the American

Correspondence: M.M. Roberts.
Received 21 February 1984; accepted 20 March 1984.

© The Macmillan Press Ltd., 1984

Screening Programmes (Beahrs *et al.*, 1979). Our campaign, described in detail elsewhere, (Roberts *et al.*, 1984) aims to provide information about breast diseases and their treatment, includes the technique of breast self-examination and is available to women regardless of their age and status within the trial.

The main objective of the Edinburgh trial is therefore to determine the value of screening for breast cancer by mammography and clinical examination in reducing mortality from the disease in well-informed women who are initially disease free.

### The study population

### *The register*

From census data it was believed that the number of women within the city boundary and in the desired age group would be of the order of 70,000. Altogether, a total of 348 practitioners within 85 practices of differing sizes were involved. This plan excluded the 11 practices which were involved in the pilot feasibility study of screening from entry to the trial.

All the eligible general practitioners except 3 gave us permission to obtain the names, date of birth and National Health Service number of women on their lists from the local Health Board. Ethical permission was obtained from the relevant ethical committees. Following this, a register was constructed and computerised for each practice. The addresses were obtained from the GPs records on the grounds that these were more likely to be up to date. In the event, it was found that Health Board and GP registers did not always coincide, most names being on both, but some being on only one or the other. This is being reported separately (Hepburn & Lutz, in preparation). Constructing a register of this magnitude took two and a half years: continual updating and checking for errors are mandatory. The register is never completely correct, and in any case is only correct for a particular moment in time, as there is continual movement of women.

The initial register consisted of women aged 38–64 years from all the eligible practices. From this, the study population is derived, and consists of:

(i) an 'initial cohort' of women aged 45–64 who entered the trial in the initial period of entry over $2\frac{1}{2}$ years (1979–1981); and
(ii) women who come into the trial each year as they reach the age of 45, together with those who have moved into the city and joined a study practice.

Altogether, there are almost 73,000 names on the register. Some women were found to have breast cancer diagnosed previously, or were outside the age limit or their names were duplicated: these were excluded or eliminated from the study. Others were found to have a wrong address, were then traced if possible and included except when it was established that they were ineligible.

At the present time (December 1983) there are 52,253 women in the study population, with a further 14,000 still to enter as they reach the age of 45 years.

### *The randomisation procedure*

Because women enter the study only through GP registers, it was felt that randomisation of practices was more desirable from the GPs point of view than randomisation of individual women. This method leads to a *cluster* sampling of women in the population.

As mentioned briefly in the introduction, the original plan before funds were obtained for a randomised trial was to screen all women in the South District. Constraints due to time forced us to register all or most practices in the South first (1979–80) and then go on to the North District. Consequently, the 45 practices in the South were stratified by size and 15 were randomly selected for screening in March 1979 by one of us (WL). Similar stratification was carried out for the 44 practices in the North district in 1980 and 16 were randomly selected for screening. Two adjustments were made: practices operating from the same premises were given the same trial status, as the GPs requested this; GPs in two premises were unintentionally told the wrong status when they were visited and the new status was allowed to stand.

Every woman on entering the trial takes her status from that of her current GP. Subsequent changes of GP are irrelevant to status.

After statistical consideration, it was decided in 1981 to increase the screening population. A random selection of three practices, originally allocated as controls, changed status and women who had entered because of registration with these practices had their entry cancelled and then joined the screening population. Any cases of breast cancer incident in the interim period are technically exclusions from the study. These cases (6 so far) are being carefully monitored.

Apart from this, no woman has changed her initial status.

### *Entry to the study*

*Initial cohort* For the women in the *screening* population, a list of potential entrants was sent to each GP who was asked to code them as follows:

(i) Suitable for invitation for screening.
(ii) Eligible but unsuitable for invitation because they were under investigation in hospital or had severe physical or mental illness.
(iii) Already had diagnosed breast cancer.
(iv) No longer in practice or died.

In the event, doctors in 5 practices did not undertake this procedure, because of lack of time or because they considered it unnecessary.

Women in Group (i) were then sent an invitation and their survey entry date was the date upon which the letters were issued. Women in Group (ii) were not invited, but were given a survey entry date corresponding to the date that their names appeared on the list sent to their GP. Women in Group (iii) were excluded from the study and those in Group (iv) had their study entry cancelled.

Once this coding has been received, letters of invitation offering an appointment for screening were sent out to suitable women.

Women entering the study as members of the *control* population take their survey entry date from the date on which their practices were indexed. Initially, they were all given the tentative status of eligibility, but subsequently each GP was sent a list of patients who had entered the study and asked to code them in a similar way as the screening practices. However, doctors in 5 of the control practices also did not carry this out. Control women were eligible, not for screening, but to receive a leaflet about breast self-examination (BSE) from the health education campaign. Women in Groups (i) and (ii) were confirmed eligible for the randomised trial: women in Groups (iii) and (iv) were treated as for the screened population above. In this way, our intention was to check the control population in a manner similar to the screened population, particularly for cases of already diagnosed breast cancer.

*Subsequent entrants* Originally the records in the register for each practice were updated once a year. Now the process is done continually by examining the files at the Health Board for new entries and deletions. For each practice, a list is compiled annually of potential new entrants to the trial (including those reaching the age of 45 years) for the GP to check eligibility as above. This is done for both screening and control practices. For women entering the screened population, the survey entry date is taken as the day the invitation is sent out (the date of the list for those deemed eligible but unsuitable). For control women, the survey entry date is the date the list was compiled.

*Improving the accuracy of the register*

Considerable effort has been made to improve the accuracy of the study population denominator and three factors are of importance.

First, about 10% of letters are returned to us by the Post Office for various reasons. A study was carried out (Morrison, personal communication 1982) on a random sample of 263 (1 in 4 of the returned letters in the South District) to determine whether women could be traced. A new address was found for 35% of such women and they were re-invited once eligibility had been confirmed. A further 28% were found to have moved either out of the area, gone abroad or died, so that they were no longer eligible for trial entry and a small number of clerical errors (7%) were corrected. The rest were not traced; 8% had been temporary residents and in 4% further information was awaited. For 17.5% of women, no record could be found. Following this study, we established a procedure to trace the individual women by checking with the GP, the Health Board, and the General Register Office for Scotland. The same procedure was then carried out for letters which were returned following the sending of the BSE leaflet to the control women. Currently 52% of women whose letters are returned are not traced. We estimate, therefore, that the residual inaccuracy of the entire population is of the order of 5%. It is likely that this will be improved further once the flagging of records is carried out (see later).

Secondly, we have gradually eliminated duplicate names from the register. In a population of this size, it is not surprising that a number of names appear on more than one GPs list.

The principle reason is delay in transferring practice records between the GP and the Health Board (Hepburn & Lutz, in preparation). So far, 1548 duplicate names have been found: they have been eliminated and the woman's correct status allocated within the trial.

Thirdly, only women with previously diagnosed breast cancer are excluded from the study population: in order to ensure complete accuracy, medical checking will be undertaken in all cases of deaths registered due to breast cancer, to determine the date of initial diagnosis. If this occurred before the survey entry date, then the case will be excluded from the study population.

Once all these various measures to improve the accuracy have been completed, we will undertake a small retrospective study in a random sample of women in the screening and control populations to check the accuracy of the initial information.

### The screening procedure

As Edinburgh is part of the U.K. Study, women who attend for screening undergo the procedure specified in the U.K. protocol. This involves mammography and clinical examination at the initial visit, followed by annual screenings with clinical examination alone at the years 2, 4 and 6, and clinical examination plus mammography in years 3, 5 and 7. Later entrants to the study will have a diminished number of episodes of screening depending on their entry date.

The clinical examination is carried out as a standardised procedure by a doctor or nurse and we are evaluating the role of nurses in this situation. Mammography is carried out using either a Philips Mammo-Diagnost U or GEC Mammostand II machine, with Kodak Min-R screens and Agfa Gevaert Medichrome film. Radiation dosage is monitored regularly: the average total skin dose for 4 exposures is about 0.006 Gy per breast.

At the initial visit, oblique and cranio-caudal views are taken: at routine subsequent visits, only the oblique view is used. The films are read by specially trained doctors, with review of abnormal films by a radiologist (AEK, BBM) and quality control maintained by the radiologist reading a random 5% of all films. Clinical examination and mammography are assessed independently to determine the role of each as screening modalities.

We aim to see 35 women in each 3 hour session, staffed by two radiographers and two nurses. A doctor is always on duty for consultation and second clinical examination while she is reading the films from the previous day's clinic. Women are also taught BSE and encouraged to carry this out once a month between visits.

Women who are found to have an abnormality on either modality are reviewed by this system (immediate second clinical examination or radiological review). Those requiring further investigation are referred to a surgical review clinic, where they are assessed and referred for biopsy if judged necessary. Fine needle aspiration cytology (Dixon *et al.*, 1984) is now performed on all palpable lumps, mainly to obtain a diagnosis of cancer prior to hospital admission, but also to evaluate the role of cytology in the screening context. If the lesion is impalpable but seen on mammography, e.g. an area of microcalcification, then a localisation procedure is carried out prior to biopsy (Chetty *et al.*, 1983).

### Monitoring breast cancer incidence and mortality

All eligible women (unless lost to follow-up) will remain in the study population until they die. Breast cancer incidence will be determined for the whole study population, including those cases detected by screening, interval cases, those arising symptomatically in women who are invited but do not attend, and in the control population. Mortality from breast cancer and all other causes will also be determined.

All new cases of breast cancer, detected by screening and otherwise are identified through a Pathology Register set up by one of us (TJA) and through liaison with the local Cancer Registry Office and its medical officer. Currently, deaths in the Lothian region are recorded from weekly and quarterly lists provided by the General Register Office, Long-term follow-up of the study population is now being planned: it is intended that all women in the study will be flagged through the NHS Central Registry, so that subsequent events (both cancer incidence and mortality) can be monitored. Careful follow-up of women with breast cancer is carried out through the collaboration of all clinicians in the area, medical records being checked annually.

Women who move abroad are deemed lost to follow-up and censored at that point.

### Statistical aspects

As we indicated earlier, the randomisation unit was the GP practice with each individual woman taking her status from that of her current GP at the time when she entered the trial. Thus her survey entry date becomes her *date of randomisation* and her trial time is measured from that point. For practical reasons, the rules for allocating survey entry date were slightly different in the two populations with women in the screening population having a date roughly 4 weeks later than they would have had as controls but this will lead to a negligible age difference between the two.

Since the aim of the trial is to determine whether screening is effective in reducing breast cancer mortality, the main outcome of interest is death from the disease; standard methods will be used to compare breast cancer mortality rates in the whole of the screening population with that of the controls (Peto *et al.*, 1977). The problem of bias in trials of screening are well documented (Feinleib & Zelen, 1969; Prorok *et al.*, 1981). By including all cancers (not just those detected by screening) we exclude length sampling bias, by taking all women in the study population (not just attenders) we avoid selection bias and by measuring time from the survey entry date we avoid lead time bias. The analysis can and will be stratified by age at entry, but unlike a clinical trial in which diagnosis precedes randomisation, it cannot be stratified by any variable measured at diagnosis. It will not be possible to stratify for any other epidemiological or demographic factors since they are recorded only for cancer cases and in those women who attend for screening.

We shall also seek to quantify the benefit of screening in terms of both years of life saved and the steady state reduction in annual mortality rates were the population to be screened continually and not for just a few years. We also plan to examine the return to normal mortality rates after the termination of screening. Other studies will include the examination of the relevance of risk factors using the prospective data routinely collected. The actual cost of screening and breast cancer treatment is being studied independently and will be reported in due course (Fraser & Clarke, personal communication 1983).

Using OPCS and GRO data for mortality and breast cancer incidence, we estimate that under the null hypothesis of no screening effect there would be 180 breast cancer deaths in the initial cohort in its first 7 years of follow-up. The HIP study results (Shapiro *et al.*, 1982) indicate that we may expect a mortality reduction of 35% demonstrable after 3–4 years and continuing until perhaps 3 years after screening ends with a decreasing percentage reduction thereafter. The present trial has a power of 83% of detecting such a reduction at the first analysis which is planned after 7 years of follow-up and a power of 95% of detecting a similar reduction after 10 years of follow-up ($\alpha = 0.05$, 1-sided test).

The women were randomised and entered sequentially by GP practice 'clusters' over a period of 2.5 years for the initial cohort, and over each subsequent year for new entrants. Even if we assume the randomisation to have been effective, then any comparison and any stratification must be of like with like and hence, for example, the initial cohort must be treated as a whole. This assumption should be checked, particularly in view of possible differences reported in a random sample of non-attenders (Maclean & Sinfield, 1983. Women who decline screening. *Report to Health Services Research Committee*, In preparation). We plan to compare the distribution of 'non-existing' women in the two populations and hence the accuracy of the denominators from which our rates are calculated.

The trial is 'pragmatic' so that protocol deviants retain their original status. Women in the control population may enlist with other screening services; women in the screening population choose whether to attend screening. Duplicate records on the register are inevitably treated differently: one wrong screening record will lead to a screening invitation, but one wrong control record does not leave a woman uninvited; for the purpose of analysis, these women are classified according to their correct status even though for ethical reasons those who have inadvertently been invited for screening continue to be routinely invited; the numbers involved are very small (at present we are aware of only 6).

It is apparent that women in the screening population have more opportunity to reveal themselves as exclusions than do the controls (a clinic attendance will reveal a previous mastectomy). However, checking the date of diagnosis for all breast cancer deaths will apply equally to both populations and thus any bias in mortality figures will be avoided.

It should also be mentioned that the register in the South District of Edinburgh was constructed using the GP records although those that did not match with Health Board records were re-checked. For the North District the register contains only records where GP and Health Board match (though discrepancies are recorded and checked). Differences in the two procedures are revealed by the fact that the 'wrong address' rate is higher in the South District (Hepburn & Lutz, in preparation). It must be emphasised however that in each area controls and screening population were treated in an identical way; therefore any possible bias can be avoided by stratifying by district (at entry).

We conclude that the randomised trial in Edinburgh has a good chance of reaching a useful conclusion about the value of breast screening and will also provide important additional evidence in the multi-centre British study.

We are grateful to the Cancer Research Campaign who made the randomised study possible with their generous support (Grant No. 1575 to MMR) and to the Scottish Home and Health Department who finance the screening programme in Edinburgh. Our thanks also go to Sir Richard Doll, Dr Jocelyn Chamberlain and Richard Peto who encouraged and supported us throughout.

The Screening Project Committee in Edinburgh consists of the following members – Dr T.J. Anderson, Dr M.M. Andrew, Professor J. Best, Dr C. Brough, Dr W. Forbes, Professor A.P.M. Forrest (Chairman), Dr R. Gruer, Dr A. Huggins, Dr A.E. Kirkpatrick, Dr N.B. Loudon, Mr W. Lutz, Dr U. Maclean, Dr M.M. Roberts and Mr J.G. Duncan (Project Administrator). Our thanks are due to this committe who were instrumental in establishing this study and have consistently given it their help and advice.

We are extremely grateful to the staff of the General Register Office Ladywell House, Edinburgh, to Dr Jennifer Webb and other staff at the Common Services Agency, to the Lothian Health Board and to the Cancer Registry Staff, (especially Mrs K. Aitkenhead) for their help and support.

We record our thanks to the members of staff of the Breast Screening Clinic, Medical Computing and Statistics Unit, Pathology and Surgical Departments without whom this work would not be possible.

Last, but not least, we thank all the General Practitioners in the city for their collaboration and support.

### References

ANDERSSON, I., ANDREN, L., HILDELL, J., LINELL, F., LJUNGQUIST, U. & PETERSSON, H. (1979). Breast cancer screening with mammography. *Radiology*, **132**, 273.

BAKER, L.H. (1982). Breast cancer detection demonstration project: Five year summary report. *Cancer*, **32**, 4.

BEAHRS, O.H., SHAPIRO, S., SMART, C., McDIVITT, R.W. (1979). Summary report of the working group to revise the NCI–ACS BCDDP's. *J. Nat. Cancer Inst.*, **62**, 642.

CHAMBERLAIN, J., CLIFFORD, R.E., NATHAN, B.E., PRICE, J.L. & BURN I. (1979). Error rates in screening for breast cancer by clinical examination and mammography. *Clin. Oncol.*, **5**, 135.

CHETTY, U., KIRKPATRICK, A.E., ANDERSON, T.J. & 4 other. (1983). Localisation and excision of occult breast lesions. *Br. J. Surg.*, **70**, 607.

DE WAARD, F. (1978). Screening for breast cancer. *Brit. Med. J.*, ii, 178.

DIXON, J.M., ANDERSON, T.J., LAMB, J., NIXON, S.J. & FORREST, A.P.M. (1984). Fine needle aspiration cytology in relationship to clinical examination and mammography in the diagnosis of a solid breast lump. *Br. J. Surg.*, (In Press).

EDINBURGH BREAST SCREENING CLINIC (1978). Screening for breast cancer. *Br. Med. J.*, ii, **175**.

FEINLEIB, M. & ZELEN, M. (1969). Some pitfalls in the evaluation of screening programmes. *Arch. Environ. Hlth.*, **19**, 412.

GEORGE, W.D. (1976). Screening for breast cancer. *Br. Med. J.*, ii, 858.

LUNDGREN, B. (1979). Population screening for breast cancer by single view mammography in a geographic region in Sweden. *J. Natl Cancer Inst.*, **62**, 1373.

PETO, R., PIKE, M.C., ARMITAGE, P. & 7 others. (1977). Design and analysis of randomised clinical trials requiring prolonged observation of each patient: II. Analysis and Examples. *Br. J. Cancer*, **35**, 1.

PROROK, P.C., HANKEY, B.F. & BUNDY, B.N. (1981). Concepts and problems in the evaluation of screening programmes. *J. Chron. Dis.*, **34**, 159.

ROBERTS, M.M., FRENCH, K. & ROBINSON, S.E. (1984). The Edinburgh Breast Education Project. *UICC Tech. Rep.*, **77**, 39.

SHAPIRO, S., VENET, W., STRAX, P., VENET, L. & ROESNER, R. (1982). Ten-to-fourteen year effect of screening on breast cancer mortality. *J. Natl Cancer Inst.*, **62**, 349.

U.K. BREAST CANCER DETECTION WORKING GROUP. (1981). Trial of early detection of breast cancer: Description of method. *Br. J. Cancer*, **44**, 618.